# Optical Fiber-Based MR-Compatible Sensors for Medical Applications: An Overview

**DOI:** 10.3390/s131014105

**Published:** 2013-10-18

**Authors:** Fabrizio Taffoni, Domenico Formica, Paola Saccomandi, Giovanni Di Pino, Emiliano Schena

**Affiliations:** 1 Unit of Biomedical Robotics and Biomicrosystems, Center for Integrated Research, Università Campus Bio-Medico di Roma, Via Álvaro del Portillo, 21, Rome 00128, Italy; E-Mails: d.formica@unicampus.it (D.F.); g.dipino@unicampus.it (G.D.P.); 2 Unit of Measurements and Biomedical Instrumentation, Center for Integrated Research, Università Campus Bio-Medico di Roma, Via Álvaro del Portillo, 21, Rome 00128, Italy; E-Mails: p.saccomandi@unicampus.it (P.S.); e.schena@unicampus.it (E.S.); 3 Institute of Neurology, Campus Bio-Medico University, and Fondazione Alberto Sordi-Research Institute for Ageing, Center for Integrated Research, Università Campus Bio-Medico di Roma, Via Álvaro del Portillo, 200, Rome 00128, Italy

**Keywords:** fiber optic sensors, MR-compatibility, interferometry, fiber Bragg grating, hyperthermia, respiratory monitoring, MR-compatible robotic assistive device

## Abstract

During last decades, Magnetic Resonance (MR)—compatible sensors based on different techniques have been developed due to growing demand for application in medicine. There are several technological solutions to design MR-compatible sensors, among them, the one based on optical fibers presents several attractive features. The high elasticity and small size allow designing miniaturized fiber optic sensors (FOS) with metrological characteristics (e.g., accuracy, sensitivity, zero drift, and frequency response) adequate for most common medical applications; the immunity from electromagnetic interference and the absence of electrical connection to the patient make FOS suitable to be used in high electromagnetic field and intrinsically safer than conventional technologies. These two features further heightened the potential role of FOS in medicine making them especially attractive for application in MRI. This paper provides an overview of MR-compatible FOS, focusing on the sensors employed for measuring physical parameters in medicine (*i.e.*, temperature, force, torque, strain, and position). The working principles of the most promising FOS are reviewed in terms of their relevant advantages and disadvantages, together with their applications in medicine.

## 1. Introduction

Historically, the first application of optical fibers to the medical field enabled the illumination of internal organs during endoscopic procedures. During the years, the same technology has been adopted to perform other tasks, such as laser treatments, and to develop transducers for monitoring parameters of interest for both therapeutic and diagnostic purposes. Although forty years have passed since the introduction of Fiber Optic Sensors (FOS) [1] and despite some advantages of FOS with respect to other mature technologies, only during the last decade has their market grown notably, thanks to the improvement of key optical components and to the decrease of the costs [2]. Currently, FOS are exploited to monitor different chemical and physical parameters of medical interests [3,4]. These sensors are commonly grouped in two categories [5]: intrinsic sensors, where the optical fiber is, by itself, the sensing element; extrinsic sensors, where the optical fiber behaves as a medium for conveying the light, whose characteristics (e.g., intensity, frequency, phase) are modulated by the measurand. Sensors of this second class allow deploying the basic components of FOS (e.g., light source, photodetector) away from the sensing element, in order to develop small size sensors and hybrid solutions [6].

In addition to the already well-established applications in industrial and medical fields, immunity from electromagnetic interferences, together with good metrological characteristics and small size, make FOS attractive for several applications that take place inside, or close to, the magnetic resonance scanner.

From the introduction of Magnetic Resonance Imaging (MRI), in the early seventies, its importance in clinical imaging has exceeded even the most optimistic hopes of researchers. The constant increase of examinations based on this technique and the introduction of new procedures performed under MRI-guidance in clinical practice have promoted research on new sensors to be applied in this scenario. Among the Magnetic Resonance (MR)-compatible exploitations, FOS can be useful both to improve surgical procedure outcomes and for patient monitoring. Examples of those applications range from the measurement of the temperature of patients undergoing MRI-guided hyperthermic procedures [7], to the assessment of deflection and force on needles during MRI-guided procedures [8], to the estimation of physiological parameters (e.g., heart rate and respiratory monitoring) [9]. Possible exploitations of such sensors for research protocols are innumerable.

The ASTM standard F2503 covers MR safety-related interactions concerning the use of devices inside the MR environment. The standard distinguishes between “MR safe”, “MR conditional”, and “MR unsafe”. “MR-safe” is an item that poses no known hazards in all MR environments; “MR-conditional” is an item that has been demonstrated to pose no known hazards in a specified MR environment under specified use conditions; “MR-unsafe” is an item that is known to pose hazards in all MR environments. Despite the ASTM subcommittee for the F04.15.11 MR Standards decided to withdraw the “MR compatible” term, it is still commonly used in medical and engineering practice. The differentiation between the terms “MR-safety” and “MR-compatibility” is crucial [10,11]. MR compatible indicates that a device, when used in the MR environment, is “MR safe” and has been demonstrated to neither significantly affect the quality of the diagnostic information, nor have its operations affected by the MR device.

In this light, fiber optic technology is particularly suitable to develop “MR-compatible” sensors, since its immunity from electromagnetic fields allows: (1) being safe; (2) not affecting image quality; (3) maintaining unaltered sensors' functionalities. Furthermore, the material used to fabricate the optical fibers does not perturb magnetic fields inside the MR-scanner, which is crucial factor for the preservation of the quality of diagnostic information.

This paper provides an overview of “MR-compatible” FOS, focusing especially on sensors employed for measuring temperature, force, torque, strain, and position during several medical procedures. Throughout the paper we offer a critical review of the most promising and widespread techniques. For the sake of clarity we arranged them in three groups: (i) FOS based on fiber Bragg grating technology; (ii) intensity-based FOS; (iii) FOS based on interferometric techniques. Moreover, measuring principles, possible medical applications, advantages and weaknesses of each method are provided and discussed.

## 2. MR-Compatible FBG-Based Sensors

### 2.1. Working Principle

MR-compatible sensors based on fiber Bragg grating (FBG) technology, developed with different possible designs, allow sensing of temperature variations and strain. The introduction of the FBG in the field of thermal and mechanical measurements started with the research of Hill *et al.*, who used electromagnetic waves to locally modify the refractive index of the optical fiber core [12]. About ten years later, the study of Meltz *et al.*, promoted the diffusion of FBGs, providing the description of a more effective, holographic technique for grating formation [13]. Thanks to the characteristics of photosensitivity technology and its inherent compatibility with optical fibers, FBGs were introduced in different fields not only related to telecommunications [14], but also to the design of FOS [15]. Despite several valuable characteristics of the FBG sensors, their spread was delayed because of the high cost and the difficulties of the manufacturing, which have been overcome only during the 1990s. The last decade saw several research groups developing sensors based on FBG. The characteristics of these sensors, their fabrication process, and their applications in medicine have been already described in detail in different reviews [4,16].

The working principle of an FBG is based on radiation reflection caused by the Bragg grating: when a fiber optic, which houses an FBG, is interrogated with a polychromatic radiation, only a narrow range of wavelengths are reflected by the FBG. The central wavelength of such range, called Bragg wavelength (λB) can be expressed as a function of the effective refraction index of the core (ηeff) and of the spatial period of the grating (Λ) as follows:

(1) $$\lambda_{B}=2\cdot \Lambda \cdot \eta_{\text{eff}}$$

The influence of both temperature and strain on Λ and η_eff_ allows the design of sensors for monitoring temperature and strain, and other physical parameters related to them (e.g., pressure, force, vibrations, and flow). Specific solutions for FBG-based transducers can be adopted in order to make them selectively sensitive to strain or temperature. Usually a reference FBG is added to the main sensor in order to attenuate the influence of undesired effects, thus improving the repeatability of the measurement system [17].

FBG technology allows the development of sensors with good metrological characteristics, such as good accuracy, large bandwidth, large dynamic range, and high strain and thermal sensitivity (typical values range from 0.64 pm/με to 1.2 pm/με, and from 6.8 pm/°C to 13 pm/°C, respectively). Moreover, this technology offers the advantages of multiplexing, because it is possible to write multiple gratings with different Bragg wavelengths on a single fiber. On the other hand, the measurement chain should adopt an expensive device to detect the wavelength of the reflected radiation (*i.e.*, an optical spectrum analyzer), in order to avoid the decrease of performances (e.g., resolution and accuracy).

### 2.2. Medical Applications

The main characteristics that make FBG technology particularly suitable for applications in medicine are biocompatibility, wide bandwidth, and small size. Furthermore, the immunity of fiber optics to electromagnetic fields, and the negligible interference with the electromagnetic fields used in MRI, make this technology very attractive for developing “MR-compatible” sensors.

**Figure 1.**
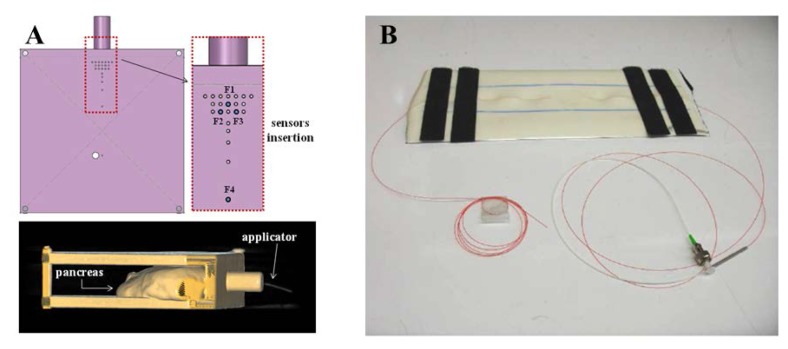

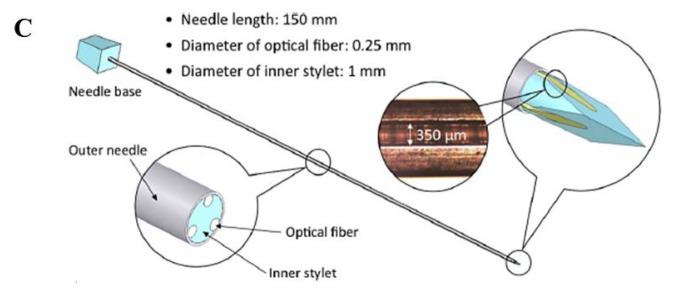
(**A**) FBG used to monitor temperature increase during hyperthermia. Both sensors and mask used to introduce them within the tissue are MR-compatible [23]; (**B**) Picture of the prototype used in [32] for respiratory and heart rate recording; (**C**) Prototype design with three embedded fiber Bragg grating sensors to measure needle deflection during MRI-guided interventions [37].

Some research groups have proposed FBG-based sensors for monitoring temperature in MRI, which is of main importance for different applications: for example Rao and colleagues developed a measurement chain for cardiac output estimation, with resolution of 0.2 °C and accuracy of 0.8 °C [18]. This technology is also employed to measure tissue temperature during MRI-guided hyperthemic treatments. Temperature plays indeed a crucial role during hyperthermia, and its monitoring can be useful to drive the physician in the adjustment of thermal exposure. However, the metrological performance of commonly used temperature sensors are affected by the electromagnetic fields used during the procedure to induce hyperthermia (e.g., the artifacts due to self-absorption of thermocouples [19]. To overcome this problem, Webb and coworkers proposed a measurement system with five FBGs, that allows them to perform temperature measures during hyperthermia treatment of kidney and liver in alive rabbits [20]. Similarly, other groups assessed the feasibility of using FBGs for temperature monitoring in swine pancreatic tissue undergoing hyperthermia [7,21,22]. In a subsequent study the same authors, in the attempt to improve spatial resolution, measured tissue temperature using 12 small size FBGs (1 mm length) to improve [23], and adopted an *ad hoc* designed MR-compatible polymethylmethacrylate (PMMA) mask to precisely arrange the optical applicator and the FBGs inside the tissue, as shown in Figure 1A. This technology has been also used to monitor temperature during cryosurgery of prostate [24] and liver [25], where the MRI compatibility was experimentally assessed.

The second field of application of FBGs in MRI is the monitoring of strain and all related parameters. During the last decade, several studies focused on the monitoring of ventilatory movement and of the respiratory rate by FBG sensors [26]. More recently, Witt and Colleagues proposed for monitoring respiratory movements a system equipped with different FOS, and an FBG-based sensor to measure thorax circumference changes [27].With a similar purpose, De Jonckheere and colleagues designed two MR-compatible sensors for recording both thoracic and abdominal movements in anesthetized patients during MRI examination. In particular, they adopted an FBG-based sensor embedded into an elastic bandage to measure thoracic movements, because of their high sensitivity to strain (*i.e.*, 1.21 pm/με) [28,29]. Grillet *et al.*, designed three sensors embedded into medical textiles for respiratory monitoring in MRI environment [30,31]; one of these sensors is based on FBG. A similar approach was adopted in Silva *et al.* to monitor both respiratory and heart rate (see Figure 1B [32]), and in Dziuda *et al.*, who assessed the feasibility of using FBG sensors for respiratory monitoring and heart activity inside a 1.5 T MRI scanner [9].

Recently large research efforts have been devoted to the introduction of FBG sensors in minimally invasive surgery. In this scenario the FBG sensors are useful to provide feedback information on the force applied to the tissue of the patient, in order to avoid damaging tissues during the application of surgical knots. Song and Colleagues developed a flexible and sterilizable FBG force sensor system for minimally invasive robotic surgery with resolution of 0.1 N, measurement error lower than 0.1 N and a measurement range up to 10 N [33].

Iordachita and colleagues developed a force measurement device for retinal microsurgery enabling to estimate the interaction forces at the tool tip with resolution of 0.25 mN [34]. Monfaredi *et al.*, described the design of a compact FBG sensor (15 mm of diameter and 20 mm of height) to provide force/torque feedback during robot-assisted prostate interventions. This is able to measure axial force ranging from −20 N to 20 N with 0.1 N resolution, and torque ranging from −200 N·mm to 200 N·mm with 1 N·mm resolution [35]. Park and colleagues employed three FBG sensors to detect the deflection of needles in MRI-guided procedures (Figure 1C [36,37]). In particular, they equipped a small-gauge MR-compatible needle with an FBG sensor in order to minimize the positioning error and thus procedural complications.

Finally, in Moerman *et al.*, an “MR-compatible” soft tissue indentor, equipped with an FBG force sensor, is presented with the aim of analyzing mechanical properties of skeletal muscle tissue. The sensor was calibrated up to 15 N showing a percentage difference lower than 3.1% with respect to the reference [38]. The main characteristics (*i.e.*, measurement principle, range of measurement, sensitivity, accuracy) and the medical fields of application of the FBG sensors described so far are summarized in Table 1.

## 3. MR-Compatible Intensity-Based and Interferometry-Based Fiber Optic Sensors

### 3.1. Working Principle

**Figure 2.**
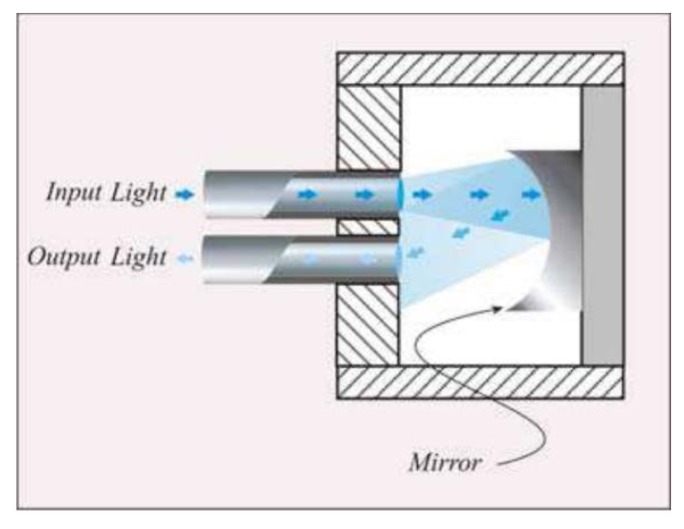
Sensing element of FOS based on intensity reflective principle: the output light is modulated by pressure or other physical parameters which cause a mirror displacement [4].

**Figure 3.**
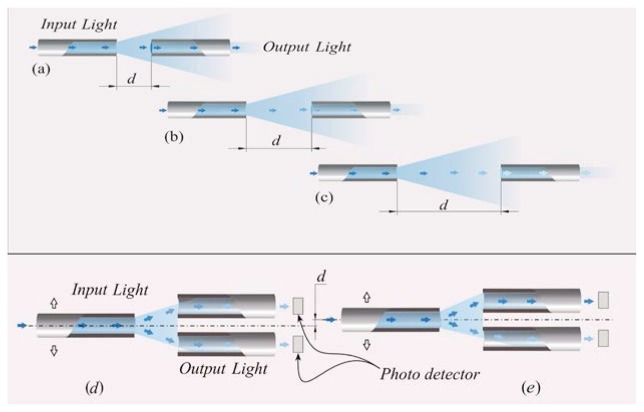
Design of an intensity-modulated FOS manufactured with two fibers. The intensity of the coupled radiation between the two fibers decreases with their distance d [4].

**Figure 4.**
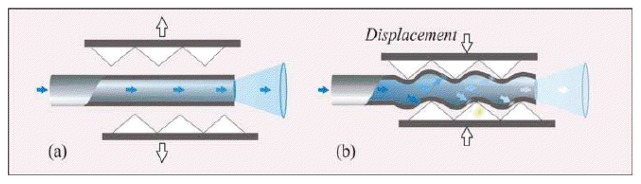
Fiber optic displacement sensors based on macrobending. The radiation intensity is modulated by the displacement of a moving part: when it does not bend the fiber (**a**); the light intensity is max0imum; on the contrary, the light intensity decreases with the bending (**b**) [4].

Different working principles based on light intensity modulation allow developing FOS for MRI applications. In this kind of sensors, the measurand modulates the intensity of light passing through the fiber. The working principles of the most widely used configurations can be grouped in three categories:

(1) Intensity reflective FOS, where a reflector (e.g., a mirror) is placed at a known distance to the distal extremities of two optical fibers, as shown in Figure 2.
  The light transported by the first fiber is reflected back by the mirror, then it is conveyed into the second fiber, which is coupled to a photodetector. In this configuration, the radiation intensity measured by the photodetector depends on the distance between the two fibers and the mirror. This configuration allows performing indirect measures since the light intensity recorded by the photodetector is related to the measurand (e.g., force, pressure, displacement, flowrate [39]) which directly acts on the mirror. Puangmali *et al.* adopted more complex configurations with the aim of improving the sensor characteristics [40].
(2) FOS based on the light coupling between two or more fibers [41]. In these sensors, the distal tip of a fiber transporting the light is placed in front of another one or a group of them. The intensity of radiation coupled to the receiving fiber(s) decreases with the relative distance between the tips, as shown in Figure 3a–c.
  FOS based on reflective and light coupling configurations are prone to unwanted drift caused by changes of input light intensity or by light lost due to fiber bending. To partially overcome these concerns, differential configurations employing two or more receiving fibers may be used (e.g., the configurations reported in Figure 3d,e). In these configurations, the outputs of two or more photodetectors are influenced in the same way by the drift. Therefore, the adequate processing of photodetectors' output allows compensating or reducing measurement errors owing to undesirable drift.
(3) Macrobending FOS: their working principle is based on the light modulation owing to fiber bending. When a light ray reaches a fiber bend, the amount of radiation lost into the cladding region increases because, beyond a critical angle, the higher-order modes hitting the cladding leak out of the fiber core. This approach can indeed be employed to measure physical parameters which cause fiber bends, such as, force, torque and pressure [42]. Usually a series of small macro bends (Figure 4) are adopted in order to increase the light leakage, aiming to improve the sensor sensitivity.
  The main advantage of the intensity modulated FOS is their cheap measurement chain: differently from FBG sensors, they do not require indeed expensive devices to measure the transducer output [5].

Finally, FOS can be based on interferometric techniques, such as, Sagnac, Fabry-Perot, and Michelson interferometer [43]. These approaches allow developing both intrinsic and extrinsic FOS. In the first case, the fiber is a medium to transport the radiation, which is modulated by a sensing element placed at its tip. In the second one the fiber itself represents the sensing element where interferences, modulated by the measurand, happen.

The most common interferometric configuration employed to develop FOS is based on Fabry-Perot interferometry. Its sensing principle exploits two semi-reflective mirrors, which partially transmits and partially reflects the light transported by the fiber. The electromagnetic waves, undergoing multiple reflections, constructively and destructively interact with themselves and produce fringes. The intensity of these fringes is function of the optical path, which is related to the distance between the mirrors. Therefore, these FOS can be used as secondary elements capable of measuring the parameters that change the distance between the two mirrors (such as pressure, force, torqu).

### 3.2. Medical Applications

**Figure 5.**
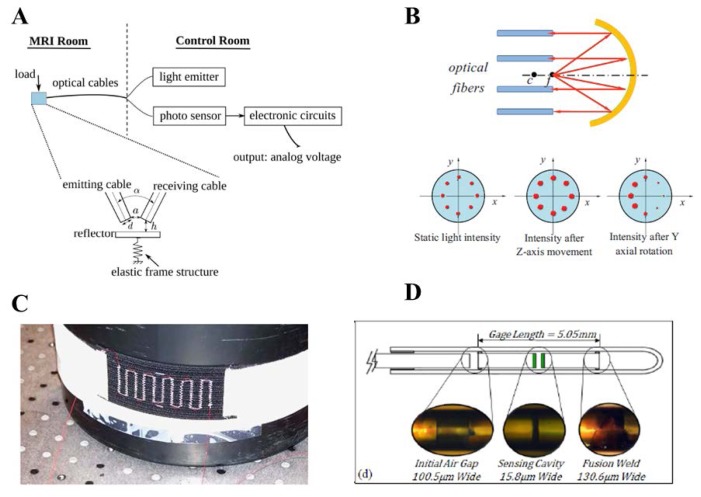
(**A**,**B**) Different configurations of intensity-based FOS tested for medical applications [46,47]; (**C**) FOS based on macrobending for respiratory monitoring [27,31]; (**D**) FOS for monitoring needle insertion force using Fabry Perot interferometry [54].

There are several examples of intensity based FOS employed in medicine. Tada *et al.*, described a simple intensity based 2-axis force sensor, where the force applied along two axes modulates the coupled light between one emission and four receiving fibers [44]. Polygerinos et al. [45] developed a three-axial force sensor based on reflective light intensity modulation. The sensor provides force feedback during MRI-guided cardiac ablation. Tan *et al.*, developed an intensity modulated and MR-compatible FOS for monitoring 3D forces during robotic MRI-guided interventions [46]. The working principle of the sensor described in this latter work is based on reflective intensity: the reflector is placed on an elastic frame structure at a distance, h, away from the transmitting and receiving fiber optics. The coupled light between the two fibers depends on the distance h, which is modulated by the applied force (Figure 5A). Gassert *et al.*, developed an “MR-compatible” robotic system equipped with a force/torque sensor and a position encoder, based on reflective intensity principle [8]. Su and colleagues developed a force/torque sensor for prostate needle placement in MRI-guided procedures [47]; the sensor is based on a spherical mirror and multiple optical fibers (Figure 5B). The light emitted from a point source is reflected by the mirror and collected by multiple optical fibers. The light intensity increases when the relative axial distance between the light source and mirror decreases. Intensity reflective FOS was also developed by Turkseven and Ueda [48] to provide force feedback in robotic applications. A similar approach was used in [49] for respiratory monitoring. The sensor measures changes of the abdominal circumference due to respiratory movements: the intensity of reflected light is modulated by the variation of the distance between the mirror and the distal part of the optical fiber, according to abdomen displacement.

Further widespread intensity-based fiber optic sensors, used in different medical applications, are based on macrobending. In Grillet *et al.*, De Jonckheere *et al.* and in Witt *et al.*, a similar approach is used to develop two macrobending sensors embedded into textiles (Figure 5C), in order to monitor respiratory abdominal movement [27,28,31]. Due to the low sensitivity compared with FBG sensors, macrobending ones are indicated for large movement monitoring. For example in respiratory monitoring, they are more appropriate for abdominal movement estimation, which are much more evident if compared with thorax excursions.

As regards interferometry-based FOS, several studies have described their use for measuring thermal and mechanical parameters of physiological interests (e.g., blood or other body compartments pressures [50–52]), but only few groups have focused their attention on MRI applications. In particular, Su *et al.*, designed and characterized a sensor for monitoring the needle insertion force during minimally-invasive prostatic surgery [53], and assessed its MRI compatibility in a 3 Tesla system [54] (Figure 5D); Liu *et al.*, developed a FOS based on low-coherence Fabry-Perot interferometry [55] for vitreoretinal microsurgery. To sum up this section, the main characteristics (*i.e.*, measurement principle, range of measurement, sensitivity, accuracy and medical applications) of intensity- and interferometry-based MR-compatible FOS are reported in Table 2.

## 4. Discussion

The introduction of MRI can be considered without any reservations as the most revolutionary milestone that has characterized the last twenty years of biomedical research and practice. Just to have an idea of its social and economic implications, the Organization for Economic Co-operation and Development (OECD) health statistics declare the presence of more than 20,000 MR scanners in the OECD countries [56], and the request for high field devices (7 Tesla or even more) is increasing worldwide.

Especially thanks to its ability to investigate and discern soft tissues, MRI has become a “cannot do without tool” in medical branches as cardiology, surgery, orthopedics and neurology. Moreover, the high spatial resolution of MRI, together with the ability of this method to indirectly obtain functional parameters of the studied tissue make it fundamental for the investigation of organ functions and for imaging-guided invasive therapy.

In the just described scenario, the need for “MR-compatible” sensors able to monitor physical parameters inside the scanner, to provide real-time feedback about the status of the patient and/or the effect produced in the tissue by surgical procedures, is growing considerably.

In this paper, we reviewed the most promising principles of work adopted to design “MR-compatible” sensors based on optical fiber technology. We devoted our attention especially to transducers developed for monitoring temperature, force, torque, strain, and position; focusing particularly on their working principles, their advantages and drawbacks, and their medical applications.

Among several possible classification criteria, we grouped the “MR-compatible” sensors according to their working principle into three classes: (i) FBG sensors; (ii) intensity-based sensors; (iii) interferometry-based sensors.

“MR-compatible” sensors based on the FBG technology have been adopted for two main purposes. First, they allow the online monitoring of the key parameters during therapeutic invasive procedures, resulting in the improvement of procedure outcomes. Among the possible examples of such applications there are: (i) the measurement and control of tissue temperature during hyperthermia or cryoablation performed under MRI-guidance; (ii) the monitoring of deflection and/or force applied on needle during MRI-guided interventions. Second, FBG have been exploited for monitoring the physiological parameters of interest (e.g., respiration and heart rate). Interferometric- and intensity-based FOS have been adopted for similar applications.

Intensity-based FOS present some concerns, mainly related to unwanted drift due to light intensity changes and bending losses; on the other hand, their measurement chain does not need complex and expensive components. For this reason they are suitable for several medical applications which not have high metrological requirements e.g., respiratory rate monitoring. The use of FBG sensors allows obtaining better sensitivity and resolution, performing multipoint measurements, lastly they are unaffected by intensity changes of input light, although they require an optical spectrum analyzer which is an expensive and bulky device. Therefore, the use of FBG technology is recommended where high performance is pivotal to improve the procedure outcome (e.g., needle deflection in microsurgery).

The introduction of FOS in clinical practice [57,58] is just at the beginning. The adequate metrological characteristics for the majority of medical applications, the absence of electrical connection with the patient, the small diameter of fiber optics are overwhelming advantages compared with the conventional transducers and motivate the market growth of this technology (e.g., FISO Technologies inc. and Camino Laboratories inc. produce pressure and temperature sensors for several medical applications). Moreover the immunity from electromagnetic field makes FOS a good candidate to meet the growing demand of MR-compatible sensors. During last years, *ad hoc* designed FOS for medical application is commercially available; e.g., Micronor Inc. and Opsense Inc. produce MR-compatible FOS for displacement, temperature and pressure monitoring.

## Figures and Tables

**Table 1.** Performances and medical applications for MR-compatible FBG sensors.

**Reference**	**Sensing Element**	**Measurand**	**Application Field**	**Characteristics**
Rao *et al.*, 1998 [18]	FBG	Temperature	Hyperthermic treatment	Accuracy ≈ 0.8 °C, range 20 °C−60 °C, Resolution ≈ 0.2 °C

Webb *et al.*, 2000 [20]	FBG	Temperature	Hyperthermic treatment	Resolution ≈ 0.2 °C

Saccomandi *et al.*, 2012–2013 [21,22]	FBG	Temperature	Hyperthermic treatment	Range up to 80 °C

Schena *et al.*, 2013 [23]	FBG	Temperature	Hyperthermic treatment	Range 20 °C−80 °C, sensitivity ≈ 8.4 pm·°C^−1^

Gowardhan *et al.*, 2007 [24]	FBG	Temperature	Cryotherapy	Minimum value ≈ −60 °C

Samset *et al.*, 2005 [25]	FBG	Temperature	Cryotherapy	Range −195 °C−100 °C

Weherle *et al.*, 2001 [26]	FBG	Inspiratory volume	Respiratory monitoring	Range 60 mL–500 mL, Frequency up to 10 Hz

Witt *et al.*, 2012 [27]	FBG	Thoracic movements	Respiratory monitoring	/

De jonckheere *et al.*, 2007 [28]	FBG	Strain	Respiratory monitoring	/

D'Angelo *et al.*, 2008 [29]	FBG	Strain	Respiratory monitoring	/

Grillet *et al.*, 2007–2008 [30,31]	FBG	Strain	Respiratory monitoring	Strain up to 41.2%, Sensitivity ≈ 0.35 nm·%^−1^Accuracy ≈ 0.1%

Silva *et al.*, 2011 [32]	FBG	Respiratory/heart rate (HR)/(RR)	Respiratory and cardiac monitoring	

Rao *et al.*, 1998 [18]	FBG	Temperature	Hyperthermic treatment	Accuracy ≈ 0.8 °C, range 20 °C−60 °C, Resolution ≈ 0.2 °C

Ioarchita *et al.*, 2009 [34]	FBG	Force	Microsurgery	Range lower than 3 mN, Resolution 0.25 mN

Dziuda *et al.*, 2013 [9]	FBG	Respiratory/heart rate (HR)/(RR)	Respiratory and cardiac monitoring	Accuracy RR: 1.2 bpm, Accuracy HR: 3.6 bpm

Song *et al.*, 2011 [33]	FBG	Force	Robotic surgery	Range up to 10 N, Resolution ≈ 0.05 N, error <0.1 N

Monfaredi *et al.*, 2013 [35]	FBG	Force/Torque	Prostatic surgery	Range −20N−20N, Resolution = 0.1N, Range −200 Nmm−200 Nmm, Resolution = 1 Nmm

Park *et al.*, 2008 [36,37]	FBG	Needle deflection	MRI-guided procedures	error in needle local curvature < 2.14%

Moerman *et al.*, 2012 [38]	FBG	Force	Tissue mechanical properties analysis	Range up to 15 N, error < 0.043 N

**Table 2.** Performances and medical applications of “MR-compatible” intensity- and interferometry-based FOS.

**Reference**	**Sensing Element**	**Measurand**	**Application Field**	**Characteristics**
Tada *et al.*, 2002 [44]	Intensity-based	Force	General purpose	Accuracy < 0.3 N, range up to 16 N

Polygerinos *et al.*, 2013 [45]	Intensity-based	Force	Cardiac ablation	Range up to 0.5 N, Resolution about 0.01 N,

U-Xuan Tan *et al.*, 2011 [46]	Intensity-based	Force	Robotic surgery and biopsy	Accuracy < 0.7 N, range up to 6 N

Gassert *et al.*, 2006 [8]	Intensity-based	Torque	MR-compatible robotic assistive device	Range ±10 Nm, resolution 0.005 Nm, sensitivity 0.66 V/Nm

Hao Su *et al.*, 2009 [47]	Intensity-based	Force-Torque	MRI guided interventions	Range up to 10 N, sensitivity ≈ 0.2 V/N

Yoo *et al.*, 2010 [49]	Intensity-based	Abdominal movement	Respiratory monitoring	/

Turkseven *et al.*, 2011 [48]	Intensity-based	Force	Robotic surgery	/

Grillet *et al.*, 2008 [31]	Macrobending	Abdominal movement	Respiratory monitoring	Range up to 3%

De jonckheere *et al.*, 2007 [28]	Macrobending	Abdominal movement	Respiratory monitoring	/

Witt *et al.*, 2012 [27]	Macrobending	Abdominal movement	Respiratory monitoring	Linear up to 5% of elongation with sensitivity of 3 mV/%

Su Hao *et al.*, 2011 [53,54]	Interferometry-based	Force	MRI guided interventions	Range up to 9.8 N, Sensitivity 40 mV/με

Liu *et al.*, 2012 [55]	Interferometry-based	Force	Microsurgery	Lateral force: Range up to 6 mN Sensitivity 40 nm/mN
